# Supplementary figures and images for: Physical Characteristics of and Transient Response from Thin Cylindrical Piezoelectric Transducers Used in a Petroleum Logging Tool
Source: Micromachines (Basel). 2019 Nov 22;10(12):804. doi: 10.3390/mi10120804 (PMC6952871; doi:10.3390/mi10120804)

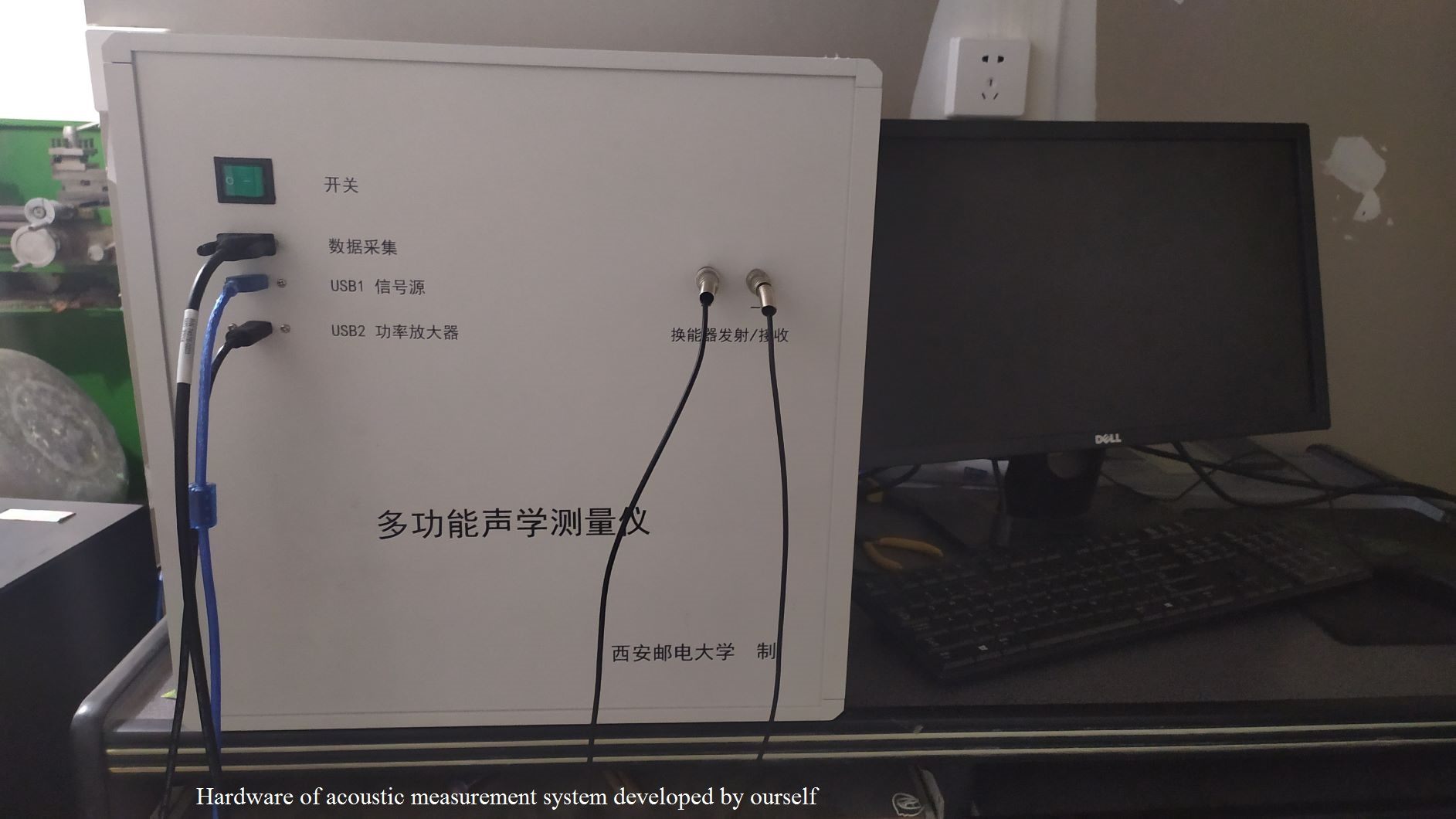

Supplement: Supplementary file 1 [file micromachines-10-00804-s001.zip › Figure S1 Hardware of acoustic measurement system.tif]

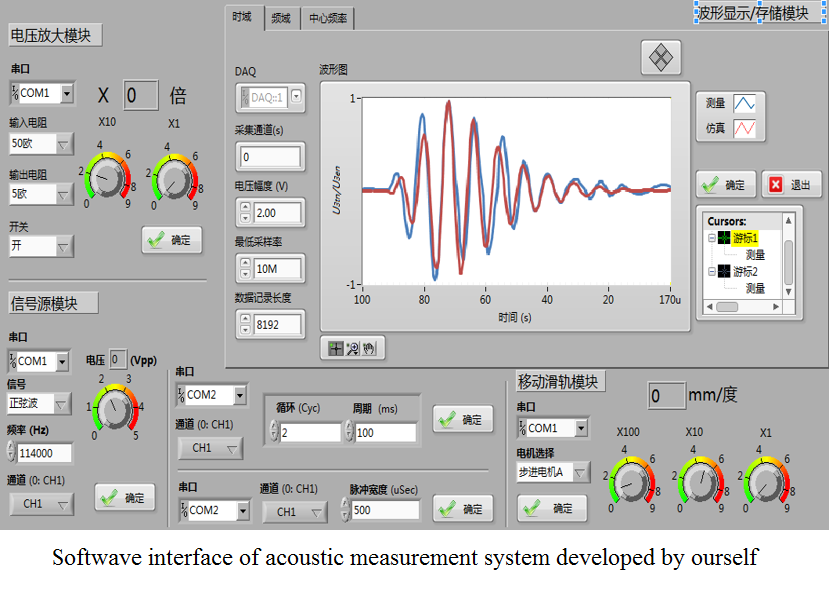

Supplement: Supplementary file 1 [file micromachines-10-00804-s001.zip › Figure S2 Softwave interface of acoustic measurement system.tif]
